# Responses to Intermittent Light Stimulation Late in the Night Phase Before Dawn

**DOI:** 10.3390/clockssleep1010004

**Published:** 2018-09-27

**Authors:** Sevag Kaladchibachi, David C. Negelspach, Fabian Fernandez

**Affiliations:** 1 Department of Psychology, University of Arizona, Tucson, AZ 85724, USA; 2 Department of Neurology, University of Arizona, Tucson, AZ 85724, USA; 3 BIO5 Institute, University of Arizona, Tucson, AZ 85724, USA

**Keywords:** light, circadian, intermittent, photostimulation, phototherapy, advance zone

## Abstract

The circadian clock is comprised of two oscillators that independently track sunset (evening) and sunrise (morning), though little is known about how light responses differ in each. Here, we quantified the morning oscillator’s responses to 19 separate pulse trains, collecting observations from over 1300 *Drosophila* at ZT23. Our results show that the advances in activity onset produced by these protocols depended on the tempo of light administration even when total exposure was conserved across a 15-min window. Moreover, patterns of stimulation previously shown to optimize the evening oscillator’s delay resetting at ZT13 (an hour after dusk) were equally effective for the M oscillator at ZT23 (an hour before dawn), though the morning oscillator was by comparison more photosensitive and could benefit from a greater number of fractionation strategies that better converted light into phase-shifting drive. These data continue to build the case that the reading frames for the pacemaker’s time-of-day estimates at dusk and dawn are not uniform and suggest that the “photologic” for the evening versus morning oscillator’s resetting might be dissociable.

## 1. Introduction

The metazoan circadian clock has long been envisaged as a complex pacemaker built atop *two* mutually coupled oscillators [1,2,3]. The first component, an evening oscillator, is said to track twilight progression at dusk to synchronize with sunset and be primarily (but not completely) responsible for engineering phase delays in response to light exposure in the early part of the night [1,4,5]. A second morning oscillator—in complement to the first—tracks a reciprocal twilight progression at dawn to synchronize with sunrise and produce advance responses to light exposure in the latter half of the night [1,4,5]. Because they can phase-lock separately to dusk and dawn and adopt different, stable phase relationships to one another, the pacemaker’s constituent oscillators provide a cogent explanation for how the circadian system is capable of measuring and adapting to seasonal variations in daylength as well as latitude-dependent changes in the photoperiod [6].

Though the dual-oscillator model has yet to be proven outright, considerable evidence suggests that it is applicable to the functional organization of the pacemaker in humans and other animals. In principle, the human circadian clock entrains to the East-to-West continuum of dawn and dusk created by Earth’s rotation about its axis [7,8]; this entrainment is conspicuous in the average geographical distribution of chronotypes, which remain tightly coupled to the solar cycle even in the presence of everyday social cues provided by work, school, friends, and family [8]. Dusk versus dawn’s biological relevance vis-à-vis circadian timekeeping can be further studied in the laboratory by housing individuals under an electric light schedule with defined transitions between lights-off and lights-on. When these boundaries are moved by shortening or extending the duration of the daily light period, the onset of nightly melatonin secretion, along with day-to-night changes in body temperature (high → low) and sleep propensity (low → high), reentrain to the new lights-off transition marking sunset [9]. Conversely, the *offset* of melatonin secretion and *night-to-day* changes in several physiological indices reentrain to the new lights-on transition marking sunrise [9]. Separate but coordinated entrainment of these ensembles would be accounted for by an evening (E) and morning (M) oscillator operating in tandem to generate seamless undulating shifts between biological night and day [10]. The presence of such oscillators in humans is hinted at more directly by the overall shape of the melatonin profile, which exhibits two daily peaks in secretion [11], and within the bimodal sleep patterns that emerge in people living under short photoperiods with forced exposure to 14 h of complete darkness [12].

The dual-oscillator hypothesis has been made more tangible in recent decades by studies elucidating an impressive hierarchy of neural and molecular substrates underlying bimodal circadian rhythms in rodents and *Drosophila* [13]. The single daily band of locomotor activity exhibited by rodents under a standard photoperiod (e.g., 12:12 light-dark (LD)) will split into two components situated about 12 h apart when the animals are continuously exposed to light for several weeks [1]. Coincident with this split in locomotion is an equal partitioning of other circadian rhythms in food and water consumption, endocrine function, and body temperature regulation [14,15], suggesting that two oscillators, now uncoupled, are working to position unified bouts of physiology and behavior to the LD transitions of the previous lighting schedule. Lesion experiments in split hamsters indicate that the identity of these two oscillators correspond to the left and right sides of the suprachiasmatic nucleus (SCN), the mammalian circadian clock, cycling in antiphase [16,17]. Since the initial characterization of this “parent” substrate, multiple dual oscillatory systems have been uncovered in the SCN, including those organized between the SCN’s major neuroanatomical subdivisions, the dorsomedial shell and ventrolateral core [18,19,20,21], those organized along cell populations positioned across the SCN’s anterior-posterior axis [22,23], and those circumscribed within the SCN neurons themselves, which may have a means of encoding dusk versus dawn via expression of two complementary sets of circadian genes [24,25,26] (conceptually similar to how unicellular organisms might achieve dual rhythmicity, [27]). Segregation of E and M oscillators—at multiple levels within the brain’s pacemaker network—is likely a motif that has been conserved across evolution: the evening and morning peaks of locomotor activity naturally exhibited by *Drosophila melanogaster* are independently generated by oscillators residing in the dorsal and ventral clusters of lateral neurons, respectively [28,29,30]. These regions undergo dramatic remodeling of their synaptic contacts daily such that different information pathways in the brain are selectively opened and closed around the timing of dusk and dawn [31,32,33].

While appreciable effort has gone into characterizing the cellular and molecular origins of the pacemaker’s E and M oscillators, considerably less so has been given to defining the extent to which each subsystem differs in its responses to light (beyond phase directionality). This question, which has been woefully unaddressed over the past 30–40 years in particular [34,35,36], is newly relevant given recent studies demonstrating the inordinate phase-shifting effects of intermittent light stimulation [37,38,39,40,41,42,43,44,45,46]. For example, when millisecond flashes are delivered once-every-second or once-every-several-seconds to flies [40,41], common laboratory rodents [42,43,44], or people [45,46], they will produce a phase jump nearly identical to that produced by nonstop illumination over the same administration period—despite the massive difference in exposure. These data, generalizable across species as they are [47], suggest that the reading frame for the metazoan pacemaker’s time-of-day estimates at dusk and dawn is *not continuous*: instead, photic information is preferentially integrated one “syllable” at a time to generate a coherent entrainment response. Presumably, these syllables are balanced across an overlapping span of milliseconds, seconds, and minutes to optimize the ratio of photic stimulation and rest that will maximize the pacemaker’s phase-shifting drive within the half-hour duration of twilight (à la Kronauer modeling, [40,48]).

If the pacemaker’s natural reading frame is discrete, based on integrating sequential bytes of photic information, then it makes sense that the logic for phase-shifting might change depending on whether the pacemaker is attempting to locate the timing of sunset or sunrise. Theoretically, the progression of illumination changes accompanying twilight should be the same for dusk and dawn albeit reversed in order; at dusk, ambient light should gradually lose irradiance and become enriched for shorter, “bluer” wavelengths and the rate at which these events occur should mirror the rate at which ambient light emerges with sunrise and re-establishes its “redder” spectral composition [49,50]. However, two factors disturb this symmetry. One involves visual perception. By the time dusk begins to usher in the evening, the eyes of diurnal animals living above the surface have habituated to daylight illumination and are unable to adapt quickly to the nuanced changes in color that proceed as skylight fades (in some cases, perception of certain hues might be impaired or altered) [51,52,53]. This contrasts with the situation at dawn when—after several hours of darkness—many animals are left with acute night vision that makes every slight change in the color of ambient light obvious [51,52,53,54]. The net effect of this asymmetry is twofold: animals will be both more light-sensitive and color-sensitive *at dawn* relative to dusk. By extension, we can hypothesize that relative to the E oscillator the M oscillator’s phase responses to light will be triggered at lower energy thresholds and be guided by a wider palette of wavelengths.

The second factor contributing to the asymmetry between dusk and dawn—and possibly modifying the phase-shifting logic for the E versus M oscillator—is the weather. Owing to daily temperature fluctuations in the surface layer of air, wind speed increases significantly during the day and dies down at night, causing a thickening of the atmosphere (wind-borne particles, pollution) and enhanced scattering of sunlight at dusk versus dawn [53,55,56]. Because light is scattered across a greater region of the sky at dusk, the sky’s color pattern will appear duller and more chaotic as the sun sets. On the other hand, because the atmosphere at dawn is clearer than at any other time of day, the sky’s color pattern will be dominated more by focal patterns of purple, blue, followed by orange-red light that emanate from the sun as it ascends the horizon. These trends reinforce predictions about the photic and color sensitivity of the M oscillator over the E oscillator and raise at least one other general hypothesis concerning their differences. Namely, that streams of “syllables” that will impact the E oscillator are likely to incorporate more variable features of light exposure than those that will impact the M oscillator, which should respond more to ordered light regimens. 

The dynamics of sunlight in nature and the pacemaker’s ability to parse these dynamics suggest that many facets of electric light could be artificially modified to influence the E oscillator in preference to the M oscillator or vice-versa. In an effort to begin teasing apart these logistics, we previously quantified how the magnitude of the E oscillator’s phase resetting is altered by different patterns of light exposure in the early part of the delay zone bordering lights-off in a 12 h:12 h LD cycle (i.e., a photoperiod that mimics the average length of day and night across the year) [40]. Here, a continuous 15-min light signal was increasingly fragmented until it produced about a third of the delay shift observed with steady luminance at zeitgeber time 13 (ZT13; an hour after lights-off or “dusk”). The remaining exposure was then reorganized along various pulse trains with different widths and interstimulus intervals to see if certain combinations of stimulation and rest could partially or fully restore pacemaker resetting despite the deficit. The results suggested that the E oscillator responds better to certain rates of light administration over others. Given a fixed level of exposure, light is most effectively translated into the E oscillator’s phase-shifting drive when it is consolidated into bouts interspersed with periods of darkness approximately three times the length of the bouts, when intermittent pulses are separated by shorter versus longer interstimulus intervals (ISIs), or when the exposure is equally distributed to the bookends of a stimulation window [40].

In the present work, we report a parallel series of experiments surveying the M oscillator’s responses to intermittent light administration. From ZT23 to ZT23.25, within an hour before lights-on or “dawn,” a continuous 15-min light pulse was systematically whittled down until it could no longer produce a full-magnitude advance shift. Exposures that resulted in significantly less resetting were then rearranged according to a dozen separate fractionation schemes that employed either: (1) designs shown to augment the E oscillator’s responses to light presentation at ZT13 or (2) other designs that made use of observations unique to ZT23. In total, ~1300 animals spread across 19 protocols were evaluated. Our findings indicate that while the M oscillator has a lower energy threshold for phase-shifting relative to the E oscillator, both subsystems behave similarly to light introduction on the order of seconds and minutes; tempos of administration that were able to improve the reset efficacy of deficient light exposure at ZT13 were able to do so at ZT23 as well. We describe these results in more detail below and briefly touch on their relevance for treating circadian disorder.

## 2. Results and Discussion

**Figure 1:**
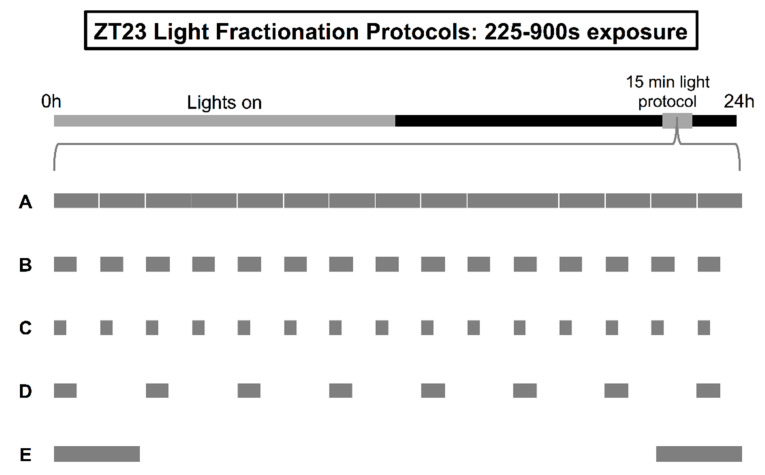
Illustration of light fractionation protocols with 225–900 s total exposure. After lights-off on the last day of a 12:12 light-dark (LD) schedule at ZT23-ZT23.25 (grey box), separate groups of flies received either a 15-min pulse of uninterrupted, constant light (**A**), or intermittent delivery of light according to the following logic: (**B**) stimulation for 30 s on the minute, (**C**) stimulation for 15 s on the minute, (**D**) stimulation for 30 s every 2 min, or (**E**) stimulation for 225 s within two symmetrical 112.5 s blocks distributed at the tail-ends of ZT23-ZT23.25.

**Figure 2:**
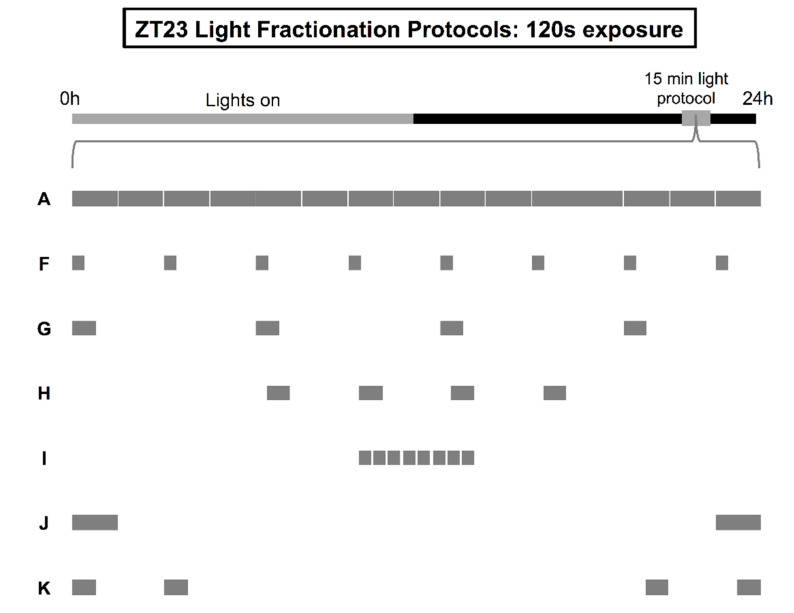
Illustration of light fractionation protocols with 120 s total exposure. After lights-off on the last day of a 12:12 LD schedule at ZT23-ZT23.25 (grey box), separate groups of flies received either a 15-min pulse of uninterrupted, constant light (**A**), or intermittent delivery of light according to the following logic: (**F**) stimulation for 15 s every 2 min, (**G**) stimulation for 30 s every 4 min, (**H**) stimulation with four 30 s pulses positioned 90 s apart, (**I**) stimulation with eight 15 s pulses positioned 15 s apart, (**J**) stimulation for 120 s within two symmetrical 60 s blocks distributed at the tail-ends of ZT23-ZT23.25, or (**K**) stimulation with two pairs of 30 s pulses (90 s ISI), each pair distributed to the tail-ends of ZT23-ZT23.25.

**Figure 3:**
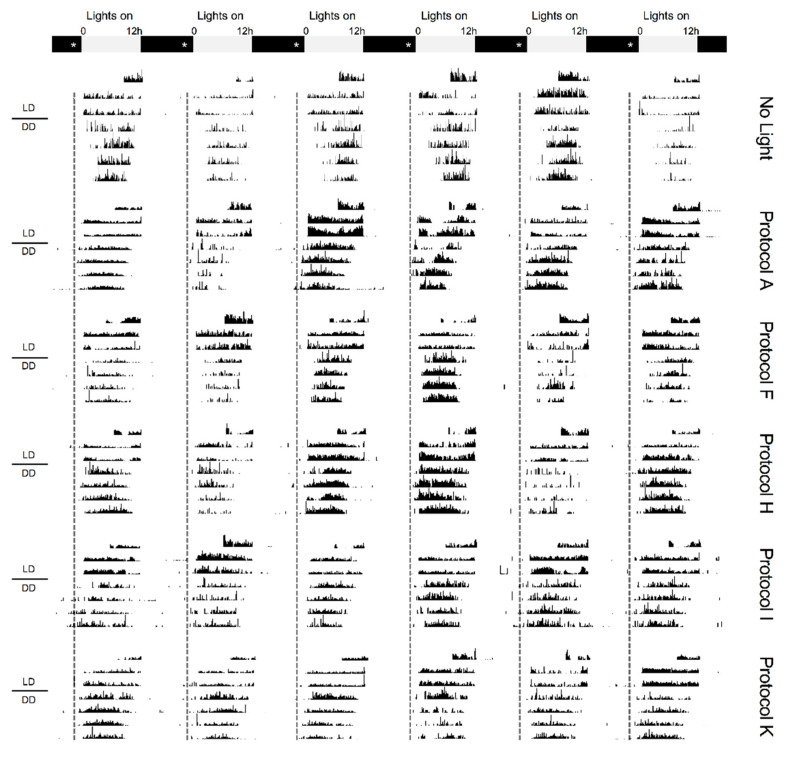
Comparing phase-shifting responses to different 120 s light fractionation regimens. Representative actograms taken from flies receiving no light (**Row 1**) or either: continuous light for 15 min (**Protocol A**, **Row 2**), 15 s of light every 2 min (**Protocol F**, **Row 3**), a series of four 30 s pulses spaced 90 s apart (**Protocol H**, **Row 4**), a series of eight 15 s pulses spaced 15 s apart (**Protocol I**, **Row 5**), or stimulation with two pairs of 30 s pulses (90 s ISI) bookending the 15-min delivery window (**Protocol K**, **Row 6**). All protocols began precisely at ZT23 and were completed by ZT23.25. Post treatment, animals were left to free-run in constant darkness (DD) for 4 days. Grey and black bars show the timing of the previous LD schedule, while asterisks and a dotted line mark the timing of light administration. Example actograms from each protocol are organized in temporally aligned columns to help visualize shifts in locomotor rhythms.

**Figure 4:**
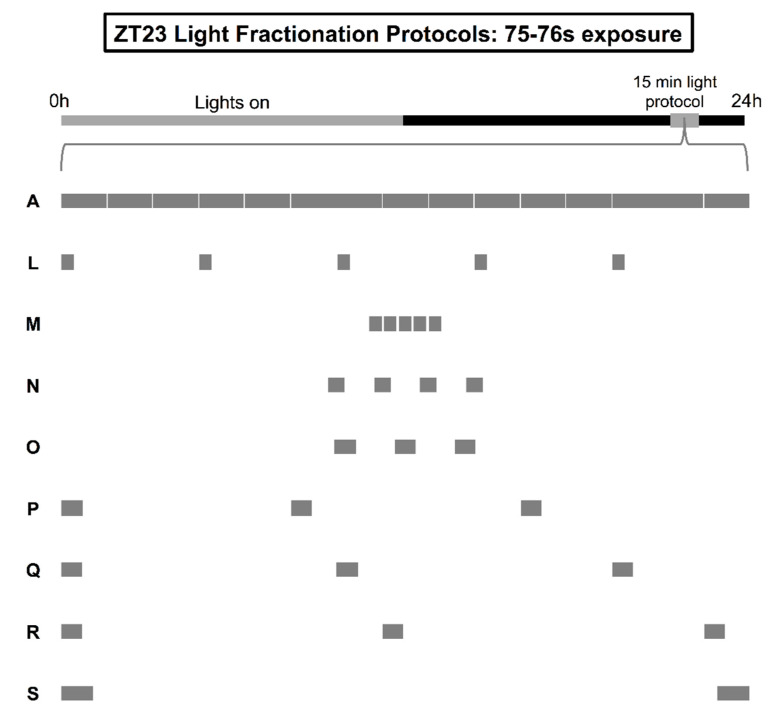
Illustration of light fractionation protocols with approximately 75 s total exposure. After lights-off on the last day of a 12:12 LD schedule at ZT23-ZT23.25 (grey box), separate groups of flies received either a 15-min pulse of uninterrupted, constant light (**A**), or intermittent delivery of light according to the following logic: (**L**) stimulation for 15 s every 3 min, (**M**) stimulation with five 15 s pulses positioned 15 s apart, (**N**) stimulation with four 19 s pulses positioned 57 s apart, (**O**) stimulation with three 25 s pulses positioned 75 s apart, (**P**) stimulation for 25 s every 5 min, (**Q**) stimulation for 25 s every 6 min, (**R**) stimulation for 25 s every 7 min, or (**S**) stimulation for 76 s within two symmetrical 38 s blocks distributed at the tail-ends of ZT23-ZT23.25.

**Figure 5:**
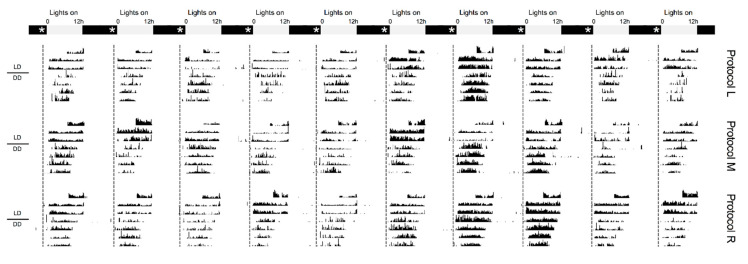
Comparing phase-shifting responses to different 75 s light fractionation regimens. Representative actograms taken from flies receiving either: 15 s of light every 3 min (**Protocol L**, **Row 1**), a series of five 15 s pulses spaced 15 s apart (**Protocol M**, **Row 2**), or light stimulation for 25 s every 7 min (**Protocol R**, **Row 3**). All protocols began precisely at ZT23 and were completed by ZT23.25. Post treatment, animals were left to free-run in constant darkness (DD) for 4 days. Grey and black bars show the timing of the previous LD schedule, while asterisks and a dotted line mark the timing of light administration. Example actograms from each protocol are organized in temporally aligned columns to help visualize shifts in locomotor rhythms.

To quantify light-induced phase resetting at ZT23, we tracked the locomotor rhythms of *Drosophila ananassae*, a particular cosmopolitan species of fruit fly that shows a unimodal pattern of locomotor activity during the day and consolidated sleep at night that mimics the diurnal sleep/wake patterns of most human adults [57,58,59]. This diurnality is not a byproduct of the LD cycle and is observed under conditions that would discourage most daytime behavior in other drosophilids [58,59]. Though *D. ananassae* do not show bimodal activity bouts centered around dawn and dusk—and as such might be less ideal models to study the E and M oscillator than a sympatric species such as *D. melanogaster*—they nevertheless offer the opportunity to study the effects of intermittent light in a context that might be slightly more applicable to humans. Similar to humans, they have already been shown to integrate photic information presented across a series of millisecond flashes and second-long pulses [40,41]. Shifts in *D. ananassae* activity onset produced by the various fractionation regimens tested in the current study are summarized in Table 1, Table 2 and Table 3, with each table organized according to one of three tiers of exposure: 225–900 s, 120 s, or approximately 75 s of 600-lux white fluorescent light. Graphical illustrations of the protocols tested at each tier are provided in adjacent figures (Figures 1, 2 and 4), along with representative actograms (Figures 3 and 5).

The phase-shifting response of the M oscillator showed surprising resilience to increasing fragmentation of a 15-min light pulse (Figure 1). The average onset shift achieved with 15 s or 30 s of white fluorescent light (600 lux) delivered on the minute for 15 min at ZT23 was statistically indistinguishable from that achieved with continuous illumination over the same period, despite a 2–4× difference in overall exposure (*F*_4308_ = 1.104, *p* = 0.3549; Table 1, protocols A–C). This resilience raised the possibility that we could repackage the 225 s content of the 15 s “duty cycle” into stimulus patterns that might evoke even larger advances than those set in motion by uninterrupted light. Previous experiments of the E oscillator at ZT13 pointed to at least two strategies that had the potential to enhance the capacity of the M oscillator’s resetting: condensing the individual 15 s pulses into 30 s signals separated by proportionally larger 90 s relaxation intervals (protocol D, i.e., 30 s of light administered every 2 min; exposure generally, but not *precisely* conserved) and redistributing the stimulation equally to the two poles of the 15-min window between ZT23 and ZT23.25 (protocol E, bookend stimulation paradigm; Figure 1) [40]. Neither strategy was able to break the response ceiling established with constant light (Tukey's post hoc test, *ps* > 0.51; Table 1).

To uncover a floor for the M oscillator’s resetting at ZT23, we continued to fragment the 15-min pulse so that 15 s of light was delivered every 2 min instead of on the minute (Figure 2, protocol F). Reducing the signal in this manner led to a significant loss of phase-shifting drive, such that the advances in activity onset generated by the 15-s-every-2-min protocol were approximately 55% of those generated by an unbroken pulse (*F*_6505_ = 12.15, *p* < 0.0001; Tukey’s post hoc test, *p* < 0.0001; Table 2, protocols A and F). This disparity gave us an opportunity to test if the remaining content from the 15-s-every-2-min protocol—120 s of 600-lux fluorescent light—could be rearranged so that it prompted larger magnitude responses from the M oscillator. In our first attempt at repackaging this stimulus, we once again evaluated circadian responses to more condensed patterns of delivery. As such, eight 15 s pulses were merged into four 30 s pulses separated by ISIs of either 210 s (delivery every 4 min; Figure 2, protocol G) or 90 s (protocol H). Consolidation of the exposure to 30 s administration every 4 min significantly enhanced the reset efficacy of 120 s of fluorescent light (Table 2, protocol F vs. G, *p* = 0.003), but did not bridge the gap with 15-min steady luminance at ZT23 (protocol A vs. G, *p* = 0.0026). However, positioning the four 30 s pulses closer—90 s instead of 210 s apart—completely recovered phase-shifting drive, doing so with almost 90% fewer photons than the standard 15-min regimen (protocol A vs. H, *p* = 0.982).

Next, we determined if phase resetting at ZT23 could also be optimized according to other strategies that were previously found to augment the information drive of light at ZT13 [40]. One fractionation approach simply shortened the ISI between pulses in the 15 s-every-2 min regimen from 105 s to 15 s (Figure 2, protocol I), while the other partitioned the exposure to two large blocks that bookended the window between ZT23 and ZT23.25 (protocol J). Shortening the timeframe between the eight 15 s pulses resulted in larger advance shifts statistically indistinguishable from those seen after 15 min of uninterrupted light—despite the 90% difference in energy (Table 2, protocol A vs. I, Tukey’s post hoc test, *p* = 0.0549). On the other hand, bookend stimulation with two 60 s pulses did not significantly increase the amplitude of resetting responses to 120 s light exposure beyond that noted with the 15 s-every-2 min baseline condition (protocol F vs. J, *p* = 0.1445). These data, collected at the 120 s and 225–900 s tiers, suggest that the M and E oscillators share similar photointegration methods at the second-to-minute timescale, though overall the M oscillator is more sensitive to light and can use more integration strategies to recover phase-shifting drive when light levels have dropped off. Previously, out of the three general approaches—pulse consolidation, ISI shortening, and bookend stimulation—only bookend stimulation was able to overcome deficient light exposure at ZT13 to recover the *full extent* of the E oscillator’s phase-shifting responses and this optimization required 225 s of stimulation (i.e., almost double the amount required for optimizing the M oscillator’s responses here) [40]. For the M oscillator, bookend stimulation is without statistical effect, but pulse consolidation and ISI shortening remain powerful methods to increase the efficiency with which light is translated into the pacemaker’s phase resetting.

Before moving on, we conducted one last experiment at the 120 s exposure tier to investigate a novel aspect of pacemaker photointegration: whether it was possible to enhance phase-shifting by combining two separate optimization strategies. Given its limited efficacy, we chose to modify the bookend paradigm so that each of the two 60 s pulses positioned at the tail-ends of ZT23–23.25 were split evenly into two 30 s pulses separated by a 90 s ISI (Figure 2, protocol K, effectively, a synthesis of protocols H and J). To our surprise, this simple adjustment changed the amplitude of the M oscillator’s phase resetting so that responses now exceeded those produced by the 15 s-every-2 min and original bookend protocols (Table 2, protocol K vs. F and J, *ps* < 0.039), and matched those produced by continuous illumination over 15 min (protocol K vs. A, *p* = 0.9655; Figure 3). These results suggest that a complicated operational logic underlies the pacemaker’s responses to light. It is not only one particular intermittent reading frame of fixed-length syllables with fixed-length ISIs that is considered when light is administered over seconds and minutes, but several reading frames where changing the characteristics and spacing between syllables will change the pacemaker’s estimate of twilight.

To conclude our study, we explored one final tier of exposure by paring down the 15 s-every-2 min protocol to one where light was delivered for 15 s every 3 min across the 15-min interval starting at ZT23 (Figure 4, protocol L). Despite the difference in energy (120 s versus 75 s of 600-lux white fluorescent light), the 15 s-every-3 min protocol did not underperform 15 s administration every 2 min; both patterns of stimulation produced identical advances of activity onset (i.e., 1.34 h; Table 2, protocol F vs. Table 3, protocol L). These data indicated that we had hit something of a plateau for the M oscillator’s resetting at ZT23. However, they provided no clues as to whether 75 s of exposure was enough to “invoke” optimization strategies that would increase light’s conversion efficiency into phase-shifting drive. It was possible that 75 s of light was simply too small of an exposure to parcel in a way that would be meaningful to the pacemaker’s running calculation of a phase shift.

To answer this question, we started by shortening the ISI between the five 15 s pulses within the 15-s-every-3-min protocol from 165 s to 15 s (Figure 4, protocol M). This maneuver resulted in statistically larger advances than those achieved with the 15 s-every-3 min baseline condition (*F*_8683_ = 9.473, *p* < 0.0001; Tukey's post hoc test, *p* = 0.0056), but did not enhance resetting any further (Table 3, protocol A vs. M, *p* = 0.0471). Next, we repackaged the five 15 s pulses according two different consolidation schemes where the ISI was set at 3× the pulse duration. One regimen merged the stimuli into four 19 s pulses separated by 57 s of darkness (protocol N), while the other merged them into three 25 s pulses separated by 75 s of darkness (protocol O). Neither regimen was able to elicit phase shifts greater than baseline (Table 3, protocol L vs. N and O, *ps* > 0.47). Considering this null result, we wondered what the circadian response might be of repositioning the three 25 s pulses from protocol O across wider and wider lengths of the 15-min stimulation window between ZT23 and ZT23.25. Would the M oscillator’s resetting be impacted by slightly different schedules where pulses were introduced on the order of every 5 min, 6 min, or 7 min (protocols P–R)? Interestingly, the breadth of the delivery interval factored into the size of the advance observed in this experimental series. Delivery of 25 s pulses every 7 min manufactured larger shifts relative to the 15-s-every-3-min baseline condition (protocol L vs. R, *p* = 0.0406; Figure 5), while delivery every 5 or 6 min did not (protocol L vs. P and Q, *ps* > 0.79). This finding, when interpreted alongside data from a final bookend experiment showing the lack of utility of two, tail-end 38 s pulses at ZT23 and ZT23.25 (protocol L vs. S, *p* = 0.4067), suggest that low levels of light exposure can benefit from bookend optimization strategies when the tail-end pulses are proportionally reduced to free up exposure for a “bridge” pulse in the middle of the stimulation window. Overall, data collected at the 75 s tier support the notion that the M oscillator is both sensitive *and flexible* in its use of light to reset endogenous rhythmicity; the delivery of as little as ~1 min of light can be engineered (and reengineered) to restrict or boost phase-shifting.

Precious few studies have attempted to distinguish how light differentially impacts phase resetting of the E and M oscillator. Those that have are in general agreement with our results that the M oscillator’s responses are *less energy dependent* than those of the E oscillator [35,36], but have not probed much further to look at differences in how these subsystems process other characteristics of light—conceivably a massive parameter space involving syllables (i.e., intermittent bytes of photic information) with different pulse shapes and length, intensities, color enrichment, and delivery intervals [37]. Inspired by the natural asymmetries in light and color perception that occur at dawn versus dusk [53], we quantified in the current study the M oscillator’s responses to over a dozen patterns of intermittent light administration at ZT23 (an hour before lights-on or “dawn”) and benchmarked them against a previous study from our laboratory that evaluated the E oscillator’s responses to the same protocols at ZT13 (an hour after lights-off or “dusk”) [40]. In sum, our results demonstrate that the reactions of the M and E oscillator to particular second-and-minute pulse trains are comparable: regimens of intermittent light that were able to coax larger phase shifts from smaller exposures were similarly effective in each. That said, the current work by no means offers a comprehensive look at the various complex logistics of light administration that could preferentially influence the M versus E oscillator, and other experiments will be necessary to determine if patterned light with—for example, different wavelength compositions and timing—can begin teasing apart their responses more substantially. It will be interesting to see if some of the M and E differences can be predicted *a priori* from what is currently known about the progression of twilight at sunrise versus sunset. Already this asymmetry has proven useful in predicting the relative amounts of intermittent light necessary for initiating phase shifts with application in the evening versus the early morning.

The fact that *D. ananassae* can integrate short light exposures to effect changes in circadian timekeeping raises an interesting mechanistic question. Up until publication of our recent work [40,41], the efficiency gains in phase-shifting brought about by brief bursts of intermittent light had been characterized only in rodents and humans [42,43,44,45,46]. The responsivity of the rodent (and presumably human) pacemaker to these stimulation trains is thought to occur via coordinated interactions between cones and a small population of melanopsin-bearing retinal ganglion cells, which are the main conduits for transmitting light information to the mammalian clock in the SCN [46]. The logic behind this model is as follows.

Light has two means by which to influence the firing of the intrinsically photosensitive retinal ganglion cell (ipRGC) population: it can excite these cells directly through activation of the melanopsin photopigment or indirectly through stimulation of retinal cones that project to ipRGCs. Because ipRGCs exhibit melanopsin-mediated responses that can persist for several minutes after light exposure has stopped [60], truncated episodes of light organized every several seconds or so have the potential to sustain ipRGC activity at a level similar to that evoked by steady luminance. If true, this means that the sluggish off-response of ipRGCs naturally limits the degree to which uniform light exposure can be translated into phase-shifting drive. The “flash” integration model (as articulated in [46]) makes the aforementioned assumption and suggests the added contribution of cones to this process. Cones orient preferentially to rapid temporal fluctuations of light (onsets and offsets), and as such their inputs to ipRGCs would be expected to provide recurring boosts to ipRGC firing set in motion by brief light sequences but not continuous illumination. While further experimentation is necessary, the flash integration model offers a parsimonious explanation for why mammalian circadian photoreception is optimized by intermittent light. However, it does not speak to why intermittent light would have the same efficacy in *Drosophila*, insects that express neither melanopsin nor cones.

A plausible substrate for circadian integration in *Drosophila* is cryptochrome (dCRY), a light-absorbing photopigment that is expressed in the compound eyes as well as the semi-transparent pacemaker neurons that comprise the fly brain’s master clock [61,62] and with a defined role in organismal photoentrainment [63]. Though structurally distinct, melanopsin and dCRY share many features that would be important for intermittent circadian photoreception, such as high light sensitivity and the ability to synthesize exposure to light over the course of minutes to hours [64]. If we assume that dCRY plays a role in *Drosophila* analogous to that played by melanopsin in mammals, what other photopigments might be involved in boosting the reset efficacy of brief light episodes? The candidates are many but might include rhodopsins in the compound eyes or Hofbauer-Buchner eyelets and interestingly dCRY itself, with different photopigment pools operating simultaneously in the compound eyes and pacemaker neurons [65]. Of these candidates, however, rhodopsin 7 (Rh7) might be the most attractive. Like dCRY, Rh7 functions as a light sensor in pacemaker neurons and contributes to circadian photoreception [66] (though healthy debate exists as to the *precise* localization of Rh7 in the pacemaker network and the significance of its contribution to clock synchronization, [67,68]). The *putative* co-expression of dCRY and Rh7 in *Drosophila* pacemaker cells would also present an intriguing correlation with the situation observed in the mammalian retina where melanopsin and cone opsins are positioned with one another to coordinate the activity of ipRGCs. Study of these photoreceptors—within their respective tandems and possibly in combination with other photopigments—might yield novel insights into how light can be organized to maximally impact the pacemaker and to differentiate responses between the E and M oscillators.

## 3. Materials and Methods

*Drosophila ananassae* were derived from an isofemale line maintained at the Drosophila Species Stock Center (DSSC) at Cornell University (stock # 14024-0371.16; NSF Award #1351502). The animals were reared at 25 °C in DigiTherm^®^ incubators (Tritech Research, Inc., Los Angeles, CA, USA), entrained to a 12:12 LD cycle (600 lux, white fluorescent lighting, lights-on at 07.00 h, MST), and transferred daily to generate a steady supply of offspring. For phase-shifting experiments, female flies were selected as late-stage, “pharate-adult” pupae, moved onto fresh food, and housed in groups of 5 to 6. A few days post-eclosion, animals were singly housed in Pyrex glass chambers (5 mm outside diameter, 65 mm long) containing a plug of corn flour-nutritional yeast-agar medium on one end (0.8% agar, 3.5% sucrose, 1.7% glucose, 6% fine-grained masa, 1% yeast) and a cotton fitting on the other, and loaded into Trikinetics DAM2 Drosophila Activity Monitors (TriKinetics, Inc., Waltham, MA, USA). Their motion was independently tracked by cross-sectioned infrared beams, which transmitted movement information over modem/USB to a computer acquisition software (DAMSystem-308) every 30 s.

An Aschoff Type II paradigm was used to quantify the effects of pulse fractionation on phase resetting of locomotor activity rhythms [69]. For the experiment, flies continued entrainment to the 12:12 LD schedule under which they were reared for 3 days. After lights-off on the last day of the schedule, separate cohorts were administered one of the following 19 light regimens (A–S) via software-controlled activation of the house lamp (600 lux, white fluorescent light; Tritech Research, DeviceCom3™). All protocols began precisely at ZT23 and ended by ZT23.25. Post light treatment, animals were left to free-run in DD for 4–5 days. Please note that the light source used in these experiments—a 4W cold-cathode fluorescent light tube—may produce transient dim afterglows when powering down, thus throwing off the temporal precision of the light regimens by a few seconds and introducing a little more exposure than what is otherwise specified in each protocol. 

Light regimens (225–900 s exposure)

**A.**  A uniform, uninterrupted light pulse delivered over 15 min.
**B.**  Intermittent delivery of light for 30 s each minute on the minute.
**C.**  Intermittent delivery of light for 15 s each minute on the minute.
**D.**  Intermittent delivery of light for 30 s every 2 min.
**E.**  A 225 s light stimulus delivered within two symmetrical 112.5 s blocks distributed at the tail-ends of ZT23 and ZT23.25. The first bookend pulse began precisely at ZT23, while the second pulse ended precisely at ZT23.25.

Light regimens (120 s exposure)

**F.**  Intermittent delivery of light for 15 s every 2 min.
**G.**  Intermittent delivery of light for 30 s every 4 min.
**H.**  A series of four 30 s light pulses separated by an interstimulus interval of 90 s (centered in the middle of the ZT23-ZT23.25 timeframe).
**I.**  A series of eight 15 s light pulses separated by an interstimulus interval of 15 s (centered in the middle of the ZT23-ZT23.25 timeframe).
**J.**  A 120 s light stimulus delivered within two symmetrical 60 s blocks distributed at the tail-ends of ZT23 and ZT23.25. The first bookend pulse began precisely at ZT23, while the second pulse ended precisely at ZT23.25.
**K.**  Two pairs of 30 s light pulses (interstimulus interval of 90 s in between individual pulses within each pair). The first pair began precisely at ZT23, while the second pair ended precisely at ZT23.25.

Light regimens (approximately 75 s exposure)

**L.**  Intermittent delivery of light for 15 s every 3 min.
**M.**  A series of five 15 s light pulses separated by an interstimulus interval of 15 s (centered in the middle of the ZT23-ZT23.25 timeframe).
**N.**  A series of four 19 s light pulses separated by an interstimulus interval of 57 s (centered in the middle of the ZT23-ZT23.25 timeframe).
**O.**  A series of three 25 s light pulses separated by an interstimulus interval of 75 s (centered in the middle of the ZT23-ZT23.25 timeframe).
**P.**  Intermittent delivery of light for 25 s every 5 min.
**Q.**  Intermittent delivery of light for 25 s every 6 min.
**R.**  Intermittent delivery of light for 25 s every 7 min.
**S.**  A 76 s light stimulus delivered within two symmetrical 38 s blocks distributed at the tail-ends of ZT23 and ZT23.25. The first bookend pulse began precisely at ZT23, while the second pulse ended precisely at ZT23.25.

Actogram plots reflecting the daily activity profile for each fly in a given treatment group were created by binning raw 30-s time series data of individual *D. ananassae*. Phase shifts of behavior were calculated by determining the horizontal distance between regression lines fitted through software-called activity onsets 2 days prior and 2–4 days after light administration (ClockLab Analysis Version 6, Actimetrics, Wilmette, IL, USA). Two days prior to the pulse, the activity onsets of ananassae were always phase-locked to the timing of lights-on in the LD schedule (i.e., 07.00 h, MST). Post-pulse, transients were observed for a day, but the flies’ behavioral rhythms stably reset by the second DD cycle (hence the start of the regression here). To correct for phase movements that might simply accompany transitions from LD to DD, a control group was transferred into DD without light treatment. Net calculations of onset shifts were normalized for the effects of LD schedule removal. Changes in phase-shift magnitude resulting from the various light fractionation schemes were evaluated by one-way ANOVA with Tukey’s post hoc correction. Significance was set at *p* = 0.05. In total, 1333 animals were independently evaluated to measure the effects of second- and minute-long bouts of intermittent light in the advance zone. Please note that data for the 15-min continuous illumination condition (protocol A) are reproduced from [40], which reports on a battery of experiments largely contemporaneous to the ones discussed in the current manuscript. An independent check for drift in the phase-shift values produced by this condition was done in the middle of the current experimental series, finding the values unchanged (2.49 ± 0.16 h, *n* = 42). Numbers in the tables are provided as mean ± SEM hours.

## 4. Conclusions

The response dichotomy of the pacemaker’s component oscillators should accrue more than just academic interest about the unique selective pressures prevailing around dawn and dusk that might have shaped each oscillator’s timekeeping adjustments to light. As it stands, many affective disorders have chronobiological underpinnings that abet their development. While circadian dysfunction has long been recognized as a contributing factor to the etiology of, say, seasonal depression or bipolar disorder [70,71], specific phototherapy protocols have yet to be institutionalized within the international medical community that selectively address the phase *delays* in wake-and-sleep often seen in people with seasonal depression as opposed to the wake-sleep *advances* characterizing people with bipolar disorder [72]. The need for better precision phototherapies is especially acute in the case of bipolar disorder because typical bright light administration techniques can trigger manic episodes in patients [73]. By understanding the processing streams by which light preferentially accesses the resetting behavior of the E versus M oscillator, we open the door to more patient-guided approaches that can help people stay mentally, emotionally, and physically healthy. 

## Figures and Tables

**Table 1:** Summary of phase-shifting data from light regimens with 225–900 s exposure.

ZT23 Stimulation Protocol		Light Exposure	Behavior Onset
ID	Description	Time (s)	Δ Phase Shift, h (*n*)
A	Continuous illumination (15 min)	900	2.42 ± 0.10 (92)
B	Intermittent pulse 30 out of every 60 s	450	2.39 ± 0.13 (66)
C	Intermittent pulse 15 out of every 60 s	225	2.19 ± 0.14 (67)
D	Intermittent pulse 30 out of every 120 s	240	2.17 ± 0.13 (27)
E	Two 112.5 s light pulses spaced 11 min, 15 s apart	225	2.16 ± 0.12 (61)

**Table 2:** Summary of phase-shifting data from light regimens with 120 s exposure.

ZT23 Stimulation Protocol		Light Exposure	Behavior Onset
ID	Description	Time (s)	Δ Phase Shift, h (*n*)
A	Continuous illumination (15 min)	900	2.42 ± 0.10 (92)
F	Intermittent pulse 15 out of every 120 s	120	1.34 ± 0.11 (81)
G	Intermittent pulse 30 out of every 240 s	120	1.88 ± 0.09 (99) ^a^
H	Four 30 s light pulses spaced 90 s apart	120	2.28 ± 0.11 (58) ^a,b^
I	Eight 15 s light pulses spaced 15 s apart	120	1.92 ± 0.16 (49) ^a,b^
J	Two 60 s light pulses spaced 13 min apart	120	1.76 ± 0.13 (55)
K	Paired 30 s light pulses (ISI 90 s), 10 min apart	120	2.28 ± 0.12 (78) ^a,b^

^a^ Light treatments producing significantly larger advance shifts than the baseline condition, stimulation protocol F; ^b^ Light treatments producing advance shifts not significantly different in size from those produced with 15-min continuous stimulation (protocol A).

**Table 3:** Summary of phase-shifting data from light regimens with approximately 75 s exposure.

ZT23 Stimulation Protocol		Light Exposure	Behavior Onset
ID	Description	Time (s)	Δ Phase Shift, h (*n*)
A	Continuous illumination (15 min)	900	2.42 ± 0.10 (92)
L	Intermittent pulse 15 out of every 180 s	75	1.34 ± 0.09 (102)
M	Five 15 s light pulses spaced 15 s apart	75	1.92 ± 0.11 (67) ^a^
N	Four 19 s light pulses spaced 57 s apart	76	1.67 ± 0.11 (67)
O	Three 25 s light pulses spaced 75 s apart	75	1.63 ± 0.11 (86)
P	Intermittent 25 s pulse every 5 min	75	1.36 ± 0.14 (62)
Q	Intermittent 25 s pulse every 6 min	75	1.60 ± 0.15 (60)
R	Intermittent 25 s pulse every 7 min	75	1.83 ± 0.09 (67) ^a^
S	Two 38 s light pulses spaced 13 min, 44 s apart	76	1.66 ± 0.12 (89)

^a^ Light treatments producing significantly larger advance shifts than the baseline condition, stimulation protocol L.
